# Application of “Internet+”‐Based Continuous Nursing to Improve the Nutrition‐Health Level of Pediatric Liver Transplant

**DOI:** 10.1111/nhs.70193

**Published:** 2025-08-13

**Authors:** Weiwei Zhang, Li Fu, Yanwei Ren, Lina Yang, Lixia Zhong

**Affiliations:** ^1^ Department of Critical Care Medicine, Beijing Friendship Hospital Capital Medical University Beijing China; ^2^ Department of General Surgery, Beijing Friendship Hospital Capital Medical University Beijing China

**Keywords:** caregiver pressure, continuous care intervention, Internet+, nutritional status, pediatric liver transplantation

## Abstract

To evaluate the effects of “Internet+”‐based continuous nursing interventions in patients with pediatric liver transplantation. Seventy‐six patients admitted between April 2019 and January 2021 were randomly divided into two groups (*n* = 38) using a random number table. The control group received conventional continuous nursing, whereas the observation group received “Internet+”‐based continuous nursing strategies in addition to conventional care. After 4 weeks, the observation group had a lower nutritional risk rate (21.06%) than the control group (42.11%). Scores on the Caregiver Stress Index (3.65 ± 1.29) and Self‐Rating Depression Scale (38.25 ± 4.32) in the observation group were substantially lower than those in the control group (7.84 ± 1.35 and 52.68 ± 4.15, respectively). The levels of albumin, prealbumin, total protein, and hemoglobin in the observation group were higher. Full adherence rates were also higher in the observation group (94.74%) than in the control group (73.68%). Postoperative complication rates were lower in the observation group (5.26%) compared with the control group (21.06%). “Internet+”‐based continuous nursing improves nutritional status, reduces caregiver stress and negative emotions, enhances family adherence, and lowers postoperative complication rates in patients with pediatric liver transplantation. This approach warrants further promotion and application.

**Summary**

- “Internet+”‐based continuous nursing improves postoperative outcomes: Pediatric liver transplant recipients receiving “Internet+”‐based continuity care demonstrated better nutritional status, fewer complications, reduced caregiver stress and depression, and higher adherence (94.74% vs. 73.68%) than the conventional care.
- Real‐time interaction enhances care quality: Platforms such as WeChat enabled real‐time communication, personalized guidance, and dynamic monitoring, thereby improving postoperative management efficiency.
- Further research is needed for long‐term validation: Limitations include a small sample size and short follow‐up; expanded studies are required to confirm long‐term efficacy and broader clinical applicability.

## 1 Introduction

End‐stage liver disease in children is a fatal condition with complex causes, including biliary atresia, hepatolenticular degeneration, hyperammonaemia, decompensated cirrhosis, and hepatoblastoma (Raina et al. 2024). Among these, biliary atresia is the main cause in Asia, accounting for 50%–60% of all children with end‐stage liver disease. Without liver transplantation, the prognosis of children with biliary atresia is extremely poor, and most die within 2 years of age (Kwong et al. 2024). Globally, the incidence of pediatric liver disease is increasing year by year, and liver transplantation has become the main intervention to save lives and improve long‐term prognosis (Desai et al. 2024). Data show that the 1‐ and 5‐year survival rates of pediatric liver transplantation can reach 85%–90% and over 80%, respectively (Ramkiran et al. 2020). Although liver transplantation technology has made substantial progress, there are still many challenges in postoperative care management, including the high risk of malnutrition in children, a high incidence of complications such as postoperative infection, and the complexity of postoperative antirejection drug management (Yi et al. 2024). In particular, the need for home care in patients with pediatric liver transplantation is more urgent, as the long‐term quality of life of this population after surgery depends on high‐quality continuity care.

Continuity care is a nursing service model designed based on patient needs. Its core aim is to fill the gap between inpatient and home care and help patients maintain health, reduce complications, and improve their quality of life through systematic and personalized nursing services (Zhang et al. 2022). Conventional care is usually limited to treatment and support during hospitalization, whereas continuity care emphasizes continuous services from hospital to home, providing postoperative support to patients through telephone follow‐up, regular visits and so on. Specifically, continuity care can guide family members on how to manage diet, take medicines, conduct rehabilitation training after surgery and assist in solving problems encountered during the nursing process. However, the traditional continuity care model still has substantial shortcomings in practice, such as low follow‐up frequency, limited care content and a lack of personalized plans (Sumner et al. 2022). Especially in the postoperative care of pediatric liver transplantation, family members' compliance is not high, and the quality of care varies, which directly affects children's recovery outcomes (Hossain et al. 2023).

In recent years, the “Internet+” continuous nursing model has provided new ideas for solving the above problems. “Internet+” continuous nursing uses social software such as WeChat and QQ to enable real‐time information sharing and two‐way communication through text, pictures, videos, and more. It allows for multiple follow‐up visits depending on the child's condition and provides richer, more diverse nursing content and personalized plans, including nutritional and psychological guidance, thus overcoming the time and space limitations of the traditional model (Hossain et al. 2023). Nursing staff can more intuitively guide families in postoperative management, offer timely feedback on the needs of patients and families, and provide targeted interventions. Studies have shown that “Internet+” continuity care is effective in chronic disease management and postoperative rehabilitation. For example, in the postoperative care of gynecological tumors and hip replacement in the elderly, this model has substantially improved patient compliance and rehabilitation outcomes (Wang 2020; Sun et al. 2021). In addition, through real‐time tracking and health education, the “Internet+” platform can help families of sick children reduce psychological burden and improve the scientific basis and standardization of postoperative care (Zhai et al. 2021; Zhao 2020).

Although “Internet+” continuity care has shown promising results in certain disease areas (Zhang et al. 2024; Li et al. 2023; Zhou et al. 2024), there are relatively few studies on its application in the postoperative care of pediatric liver transplantation. Patients with pediatric liver transplantation have distinct needs. Exploring the effect of “Internet+” continuity care in this population can provide improved nursing support for such patients.

Therefore, this paper compares the effects of “Internet+” continuous care and traditional continuity care to explore the practical value of this model in improving the postoperative nutritional status of children undergoing liver transplantation, reducing the stress and negative emotions of caregivers, improving nursing compliance and reducing the incidence of postoperative complications, thereby providing a scientific basis for the further promotion of “Internet+” continuity care.

## 2 Materials and Methods

### 2.1 Study Design

This study was a prospective randomized controlled trial, and the research design underwent strict ethical review by the Bioethics Committee of Beijing Friendship Hospital, Capital Medical University (ethical approval number: 2023‐P2‐378‐01). The sample size calculation was based on the primary outcome indicator—the incidence of malnutrition risk—setting *α* = 0.05 and *β* = 0.1 (test power 90%), in combination with malnutrition risk rates reported in previous literature (Tuokkola et al. 2021). A minimum of 34 children were required in each group. Considering a 10% dropout rate, the final sample size for each group was 38, with a total sample size of 76.

### 2.2 Subjects

**FIGURE 1:**
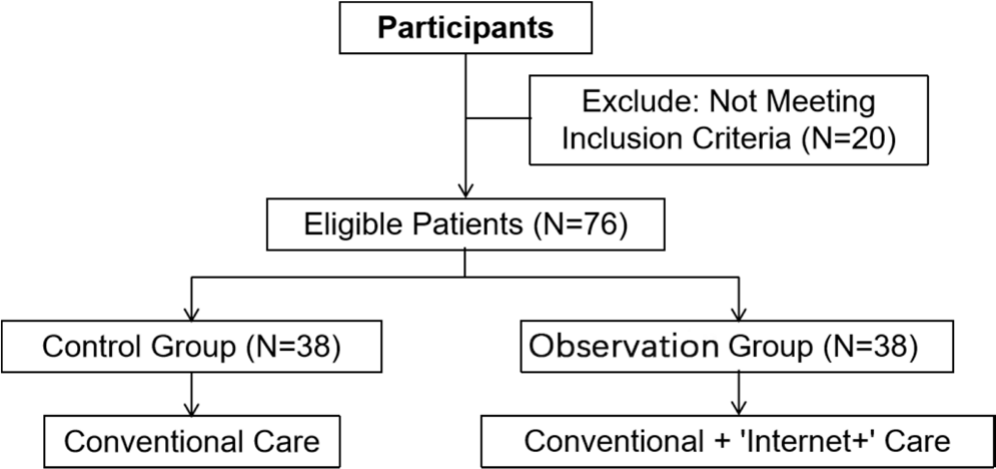
Flow chart.

A total of 76 pediatric patients who underwent liver transplantation at Beijing Friendship Hospital, Capital Medical University in Beijing between April 2019 and January 2021 were selected as study participants. The inclusion criteria were as follows: (1) all patients had received liver transplantation and survived for at least 6 months; (2) all family members were informed and signed the risk notification form; and (3) all patients had complete laboratory test reports and follow‐up data. The exclusion criteria were as follows: (1) patients with abdominal adhesions, obvious abdominal distension, or malnutrition prior to surgery; (2) postoperative haemodynamic instability or other serious complications (such as abdominal hemorrhage or intestinal adhesions); (3) family members of children with severe mental or cognitive disorders (Zhai et al. 2021; Zhao 2020). The flow chart is shown in Figure 1.

### 2.3 Grouping and Randomization

The subjects were divided into an observation group and a control group using a random number table, with 38 cases in each group. Randomization was completed by a research assistant who was not involved in patient care, and the subjects were assigned to the two groups in a 1:1 ratio. To avoid selection bias, participants were not informed of their group assignment during the nursing intervention, and the assessors of the observation indicators were also unaware of group allocation. We adopted a single‐blind design.

### 2.4 Intervention Measures (See Figure 2)

**FIGURE 2:**
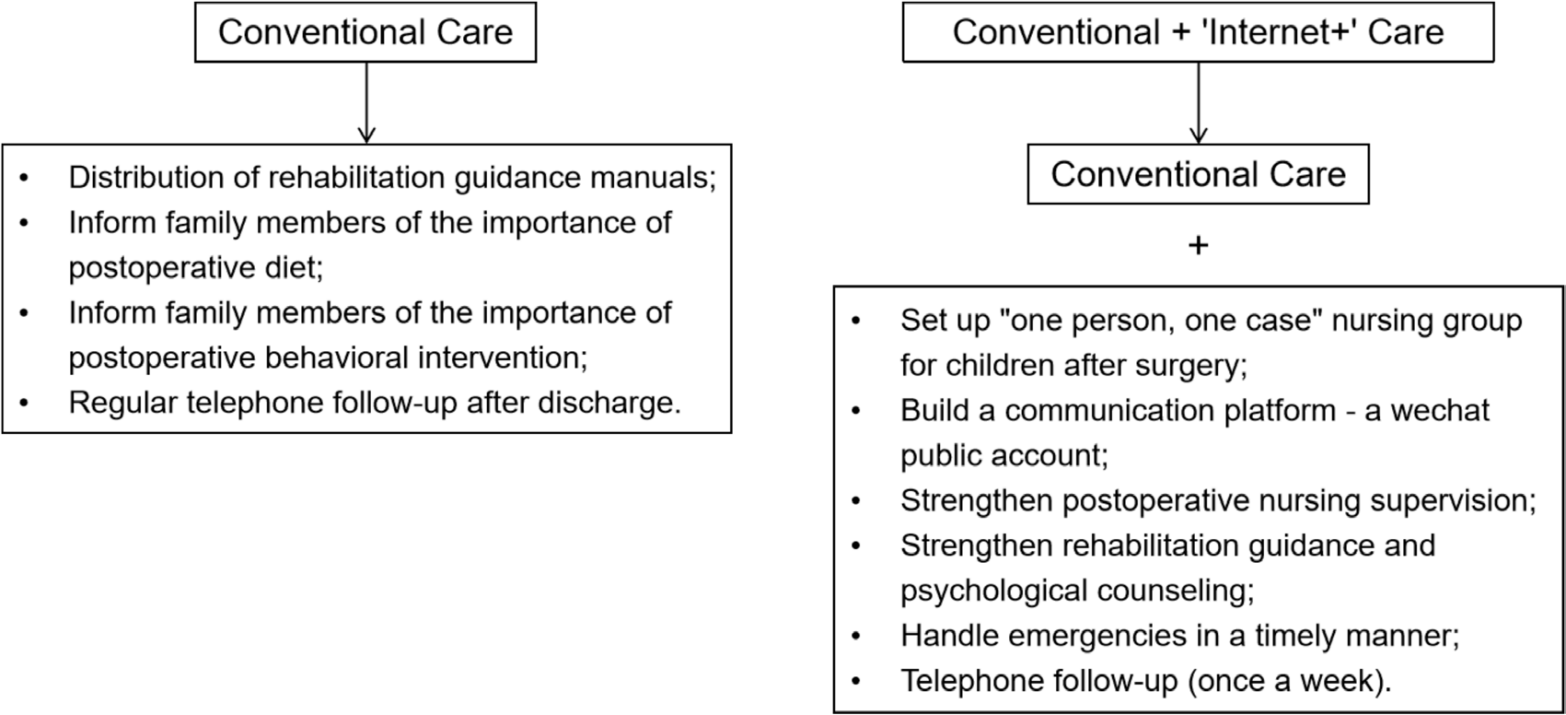
Intervention measures.

#### 2.4.1 Control Group

Routine continuity care was adopted. According to the child's condition, reasonable discharge instructions and interventions were formulated, including the following measures (He and Ren 2020):

1. Distribute the rehabilitation guidebook *New Liver Baby*. Through simple text and comics, it presents details of the rehabilitation process, such as diet after pediatric liver transplantation, pipeline maintenance, anti‐rejection drug administration and precautions, and early postoperative activities to help children return to daily life more effectively.
2. Inform the family members of the importance of the child's diet after surgery. Encourage the intake of high‐protein, high‐vitamin foods such as eggs, dried shrimp, and fruits.
3. Inform family members of the importance of behavioral intervention after surgery. Strengthen passive training for children and avoid strenuous activity. Family members should communicate with the child more frequently to help build their confidence.
4. Regular follow‐up after discharge—telephone follow‐up. Follow‐up visits are conducted by telephone using a standardized follow‐up process, with visits once a week. Specific follow‐up content includes monitoring for complications in the child, providing nursing guidance, answering questions from family members, tracking medication status, and discussing care experiences. In special circumstances, such as when the patient actively seeks help, the number of follow‐up visits can be increased, or the patient can be asked to return to the outpatient clinic for evaluation (Hossain et al. 2023).

#### 2.4.2 Observation Group

On the basis of conventional continuity care, an “Internet+”‐based continuity care intervention was carried out, mainly including the following measures:

1. Establish a postoperative “one person, one case” nursing team for the child: Team members include nursing professionals, liver transplant experts, rehabilitation experts, and psychological experts.
2. Build a communication platform: The department has established a WeChat public account, which contains relevant details of the recovery process, such as diet after pediatric liver transplantation, pipeline maintenance, antirejection drug administration methods and precautions, and early postoperative activities. The observation group, WeChat communication group, and WeChat check‐in group are established. The group administrator is an experienced nurse who has mastered various tasks after pediatric liver transplantation and manages them regularly each day.
3. Strengthen postoperative nursing supervision: This includes asking children to take photos of their meals, medications, and rehabilitation exercises in the WeChat check‐in group of the observation group. The group administrator reviews each child's diet daily to assess whether the nutrients are high in vitamins, low in sugar and fat, and include an appropriate amount of high‐quality protein; whether they eat small meals frequently; and whether the food is soft, easy to digest, low in fiber, and low in stimulation, such as soft or semiliquid food. It is also checked whether the children take medication regularly, in sufficient amounts, and correctly as directed by the doctor.
4. Strengthen rehabilitation guidance and psychological counseling: The observation group shares and exchanges videos on passive training, exercise points, communication techniques, and psychological knowledge for children in the WeChat communication group to create a positive communication atmosphere.
5. Handle emergencies promptly: The department's contact information is reserved. If the observation group encounters an emergency, a professional is contacted to handle it promptly, with follow‐up arranged during regular visits.
6. Conduct weekly follow‐up calls: Call the patient once a week to understand their recovery after surgery and provide guidance on key care points for the following week (Li et al. 2020; Ren and Luo 2015).

### 2.5 Observation Indicators

According to the purpose of the study, the outcome indicators are divided into primary outcome indicators and secondary outcome indicators:

#### 2.5.1 Main Outcome Measures

Nutritional status of children, including malnutrition risk rate and laboratory indicators.

Before and after 4 weeks of nursing, the two groups were assessed using the nutritionists' Screening Tool for the Assessment of Malnutrition in Pediatrics (STAMP) to evaluate the nutritional status of patients. STAMP was proposed by McCarthy et al. in 2004 and is suitable for hospitalized children aged 2–17 years. The tool consists of three parts: disease risk, dietary intake, and anthropometric indicators. The total score is 0–9 points, where a total score of 0–1 is low risk, 2–3 is moderate risk, and ≥ 4 is high risk (Amateau et al. 2004). The Cronbach's *α* coefficient of the scale is 0.897. STAMP's high sensitivity and high positive likelihood ratio enable it to better screen out children with nutritional risks, whereas its high specificity can reduce the screening of children who are not at nutritional risk. At the same time, because it is operated by nurses and is simple and easy (average time is 10 min), it is widely used in China.

Three milliliters of peripheral fasting blood were taken at 9:00 every other morning, and the patient's albumin (ALB), prealbumin (PA), total protein (TP), and hemoglobin (Hb) levels were measured using an automatic biochemical analyzer to evaluate the liver's synthetic function after liver transplantation (Ren and Luo 2015; Amateau et al. 2004).

#### 2.5.2 Secondary Outcome Measures

1. Caregiver stress: The two groups were assessed using the Chinese version of the Caregiver Stress Index (CSI, total score of 13 points; the higher the score, the greater the stress) before and 4 weeks after care (Zhuo et al. 2020), the Cronbach's *α* coefficient of the scale is 0.942.
2. Caregiver psychological status: Self‐Rating Anxiety Scale (SAS) and Self‐Rating Depression Scale (SDS) scales were used to assess anxiety and depression levels. Self‐rating anxiety (SAS, with a cutoff of 50 points; the higher the score, the more severe the anxiety) and self‐rating depression (SDS, with a cutoff of 50 points; the higher the score, the more severe the depression) were used to assess the stress and psychological condition of the caregivers of the children (Desai et al. 2024; Wu and Fu 2020); the Cronbach's *α* coefficient of the scale is 0.907.
3. Family awareness rate: Four weeks after the nursing care, the two groups used the family awareness questionnaire that had been verified in previous studies to evaluate the awareness of matters such as attending follow‐up visits on time, compliance with anti‐rejection drugs, and nutritional intervention. The total score of each item was 100 points, and ≥ 90 points was considered awareness (Peng et al. 2015). The Cronbach's *α* coefficient of the scale is 0.937.
4. Incidence of postoperative complications: The number of common postoperative complications such as infection, bile leakage, and vascular embolism was recorded.
5. Compliance with postoperative care and rehabilitation: The observation group regularly counted the children's postoperative care and rehabilitation compliance every week, including the following:
  – Complete compliance (the sum of the number of incorrect diet and/or incorrect medication is ≤ 10% of the total number of times);
  – Partial compliance (total number of times 10% < sum ≤ total number of times 40%);
  – Noncompliance (sum > 40% of the total number of times).

The control group was asked every week whether the children had an improper diet or medication, and details were gathered to determine whether they belonged to the full compliance, partial compliance, or noncompliance group. If the child was fully compliant for all 4 weeks, the final result was recorded as full compliance. If there was one instance of noncompliance in the 4 weeks, the child was recorded as noncompliant. The rest were considered partially compliant (Wu and Fu 2020), and the Cronbach's *α* coefficient of the scale is 0.873.

### 2.6 Statistical Analysis Methods

All data were processed using SPSS 24.0 software (IBM, Armonk, NY, USA). Descriptive statistics were used for patient demographic data, and count data were described as percentages (%). The chi‐square test (or Fisher's exact test) was used for categorical variables. Measurement data were expressed as *x̄* ± *s*, and comparisons between the two groups were conducted using the *t* test or a nonparametric test, as appropriate. Multivariate analysis was performed using logistic regression. A *p* value of < 0.05 was considered statistically significant.

In the logistic regression analysis, the outcome indicators were defined as dependent variables according to the purpose of the study, including the risk of malnutrition after 4 weeks of care (whether there was a risk of malnutrition, 0 = no, 1 = yes) and occurrence of postoperative complications (whether complications occurred, 0 = no, 1 = yes). The independent variables included the following factors: nursing mode (0 = conventional care, 1 = continuous care based on “Internet+”); malnutrition before care (0 = no, 1 = yes); awareness rate of nutrition knowledge (0 = know, 1 = unknown); patient care compliance (0 = complete compliance, 1 = partial/non‐compliance); caregiver stress (continuous variable, CSI score); gender (0 = woman, 1 = man); age at the time of surgery (continuous variable); type of liver transplantation (0 = autologous transplantation, 1 = allogeneic transplantation); and disease type (categorical variables: biliary atresia, hepatolenticular degeneration, hyperammonaemia, decompensated cirrhosis, and hepatoblastoma.). The effects of the above independent variables on the risk of malnutrition and postoperative complications were analyzed using logistic regression to identify the main intervention factors and related influencing mechanisms.

## 3 Results

### 3.1 Demographic Characteristics

A total of 76 children were included in this study, with 38 in the observation group and 38 in the control group. The vast majority of liver transplants were autologous, with only 17.11% (13/76) being allogeneic. The types of diseases were mostly biliary atresia, hepatolenticular degeneration, and hyperammonaemia, with biliary atresia accounting for as high as 55.26% (42/76), and the proportion in the control group was higher than that in the observation group. There were no significant differences in demographics and preoperative and intraoperative clinical characteristics between the two groups (*p* > 0.05), indicating that the randomization was reasonable and the two groups had a good baseline balance (see Table 1 for details).

**TABLE 1:** Demographic characteristics of the two groups of patients (*N* = 76).

Demographic characteristics		Total	Observation group	Control group	*χ* ^2^/*t*	*p*
Gender	Male	44 (57.89%)	21 (55.26%)	23 (60.53%)	0.216	0.642
	Female	32 (42.11%)	17 (44.74%)	15 (39.47%)		
During surgery Average age (years)		2.61 ± 0.62	2.62 ± 0.65	2.59 ± 0.61	0.893	0.936
BMI (kg/m^2^)		15.3 ± 1.2	15.4 ± 1.1	15.2 ± 1.3	0.418	0.678
Household income (monthly, yuan)		8000 ± 2500	7900 ± 2300	8100 ± 2700	0.311	0.756
Are there any preoperative complications (yes)		16 (21.05%)	8 (21.05%)	8 (21.05%)	0.000	1.000
Operation time (h)		6.2 ± 0.8	6.3 ± 0.7	6.1 ± 0.8	1.095	0.277
Operation blood loss (mL)		210 ± 45	215 ± 40	205 ± 50	0.936	0.352
Liver transplantation	Parent	63 (82.89%)	31 (81.58%)	32 (84.21%)	0.093	0.760
	Heterogeneous	13 (17.11%)	7 (18.42%)	6 (15.79%)		
Disease type	Biliary atresia	42 (55.26%)	19 (50.00%)	23 (60.53%)	1.072	0.899
	Wilson's disease	11 (14.47%)	6 (15.79%)	5 (13.16%)		
	Hyperammonemia	15 (19.74%)	8 (21.05%)	7 (18.42%)		
	Decompensated cirrhosis	5 (6.58%)	3 (7.89%)	2 (5.26%)		
	Hepatoblastoma	3 (3.95%)	2 (5.26%)	1 (2.63%)		

### 3.2 Nutritional Status

Based on the STAMP pediatric malnutrition assessment scale screening, as shown in Table 2, before the intervention, the proportion of malnutrition risk cases in both groups was high, accounting for 92.11% (35/38) in the observation group and 86.84% (33/38) in the control group. There was no significant difference in nutritional status between the two groups before the intervention (*p* = 0.345, *p* > 0.05). After the intervention, the number of malnutrition risk cases in the observation group was significantly lower than that in the control group, and the difference was statistically significant (*p* = 0.048, *p* < 0.05; see Table 2 for details).

**TABLE 2:** Comparison of the two groups of NRS2002 nutrition screening scales^a^ (*N* = 76).

Group	Number of cases at risk of malnutrition before nursing	Number of cases at risk of malnutrition after 4 weeks of care
Observation group (*n* = 38)	92.11% (35/38)	21.06% (8/38)
Control group (*n* = 38)	86.84% (33/38)	42.11% (16/38)
$\chi^{2}$	0.559	3.897
*p*	0.455	0.048

^a^ Fisher's exact test estimate *p* value.

Before the intervention, there was no significant difference in liver synthetic function between the two groups, and the *p* values were all greater than 0.05. After 4 weeks of nursing, the observation group and the control group had ALB (42.84, 36.79), PA (264.69, 223.22), TP (72.85, 59.26), and Hb (146.53, 136.72), respectively. The ALB, PA, TP and Hb values of the observation group were higher, but only the difference in ALB was statistically significant (*p* = 0.044). This indicates that “Internet+” continuous care has advantages in improving the nutritional status of children (see Table 3 for details).

**TABLE 3:** Comparison of nutritional status (g/L, $\bar{x}\pm s$) (*N* = 76).

Group	ALB	PA	TP	Hb
Observation group before care	34.29 ± 4.59	183.21 ± 24.69	56.23 ± 5.97	122.49 ± 13.68
Control group before care	35.31 ± 4.62	202.58 ± 24.32	53.25 ± 5.92	123.41 ± 13.72
*t*	0.965	3.445	2.185	0.293
*p*	0.511	0.180	0.273	0.819
After 4 weeks of nursing care, the Observation group	42.84 ± 2.11	264.69 ± 18.45	72.85 ± 7.39	146.53 ± 16.74
After 4 weeks of care, the control group	36.79 ± 1.47	223.22 ± 15.68	59.26 ± 6.46	136.72 ± 15.39
*t*	14.503	10.558	8.535	2.659
*p*	0.044	0.060	0.074	0.229

Abbreviations: ALB: albumin; Hb: hemoglobin; PA: prealbumin; TP: total protein.

### 3.3 Stress and Psychological Scores of Caregivers in the Two Groups

Before nursing, the CSI scores of the observation group and the control group were 9.32 and 9.46, SAS scores were 56.12 and 58.31 and SDS scores were 62.87 and 60.59, respectively. There were no significant differences in these scores. After 4 weeks of intervention, the CSI scores of the observation group and the control group were 3.65 and 7.84 (*t* = 13.832, *p* = 0.046), SAS scores were 42.41 and 50.98 (t = 11.655, *p* = 0.054), and SDS scores were 38.25 and 52.68 (*t* = 14.849, *p* = 0.043). The CSI and SDS scores of the observation group were substantially lower than those of the control group. This shows that “Internet+” continuity care can effectively alleviate the negative emotions of family members (see Table 4 for details).

**TABLE 4:** Comparison of stress and psychological scores of caregivers between the two groups (points, $\bar{x}\pm s$).

Group	CSI	SAS	SDS
Observation group before care	9.32 ± 1.97	56.12 ± 4.25	62.87 ± 5.61
Control group before care	9.46 ± 2.43	58.31 ± 3.41	60.59 ± 5.24
*t*	0.276	2.478	1.831
*p*	0.829	0.244	0.318
After 4 weeks of nursing care, the observation group	3.65 ± 1.29	42.41 ± 3.02	38.25 ± 4.32
After 4 weeks of care, the control group	7.84 ± 1.35	50.98 ± 3.38	52.68 ± 4.15
*t*	13.832	11.655	14.849
*p*	0.046	0.054	0.043

Abbreviations: SI: Caregiver Stress Index; SAS: self‐rating anxiety; SDS: self‐rating depression.

### 3.4 Comparison of Nursing Compliance, Awareness Rate, and Postoperative Complications Between the Two Groups

The rates of full compliance and partial compliance in the observation group were 94.74% (36/38) and 5.26% (2/38), respectively. The rates of full compliance, partial compliance, and noncompliance in the control group were 73.68% (28/38), 23.68% (9/38), and 2.63% (1/38), respectively. The compliance in the observation group was significantly higher than that in the control group (*p* = 0.040 < 0.05; see Table 5).

**TABLE 5:** Comparison of nursing compliance between the two groups of patients.

Group	*n*	Full compliance	Partial compliance	Non‐compliance	*χ* ^2^	*p*
Observation group	38	36 (94.74%)	2 (5.26%)	0 (0%)	6.455	0.040
Control group	38	28 (73.68%)	9 (23.68%)	1 (2.63%)		

After 4 weeks of nursing, the awareness rates of nutritional intervention knowledge in the observation group and the control group were 97.37% (37/38) and 94.74% (36/38), respectively, with no significant difference (*p* = 0.724). Among the postoperative complications, the incidences of postoperative infection, bile leakage, and vascular embolism in the control group were higher than those in the observation group, and the total incidence of complications in the control group was higher than that in the observation group (*p* = 0.042 < 0.05), as shown in Table 6.

**TABLE 6:** Comparison of the awareness rate of nutritional intervention knowledge and the number of postoperative complications between the two groups.

Group	Awareness rate of nutritional intervention knowledge	Complication			
		Postoperative infection	Bile leakage	Vascular embolism	Total
Observation group (*n* = 38)	37 (97.37%)	1 (2.6%)	0	1 (2.6%)	2 (5.26%)
Control group (*n* = 38)	36 (94.74%)	3 (7.9%)	2 (5.26%)	3 (7.9%)	8 (21.06%)
χ^2^	0.347	1.056	2.054	1.056	4.145
*p*	0.556	0.304	0.152	0.304	0.042

### 3.5 Logistic Regression Analysis of Nutritional Status

This study analyzed the nutritional status after 4 weeks of care, using an unpaired design and taking the presence or absence of malnutrition risk as a binary dependent variable (0 = no malnutrition risk, 1 = malnutrition risk). A multivariate analysis was performed using a logistic regression model to assess the independent effects of multiple single and composite independent variables on the risk of malnutrition. The analysis results showed that patient care compliance, continuity of care, and caregiver stress were the main factors affecting nutritional status (*p* < 0.05; detailed results are shown in Table 7).

**TABLE 7:** Results of logistic regression analysis of nutritional status.

Variable	Standardized coefficient *β*	OR	95% CI	*p*
Constant	—	1.753	(1.383, 2.176)	< 0.001
Patient care adherence	−0.391	0.371	(0.172, 0.799)	0.011
Continuity of care	−0.303	0.479	(0.265, 0.868)	0.015
Caregiver stress	−0.239	0.59	(0.369, 0.942)	0.027
Nutritional knowledge awareness rate	−0.210	0.810	(0.592, 0.952)	0.136
Gender	0.059	1.061	(0.936, 1.205)	0.258
Age at time of surgery	0.115	1.122	(0.992, 1.269)	0.067
Types of liver transplantation	−0.120	0.899	(0.421, 1.912)	0.784

### 3.6 Logistic Regression Analysis of Complications

The steps were the same as above. After screening using the stepwise regression method, four variables were finally included in the model: liver transplantation, continuity of care, patient care compliance, and caregiver stress (see Table 8).

**TABLE 8:** Results of logistic regression analysis of complications.

Variable	Standardized coefficient *β*	OR	95% CI	*p*
Constant	—	1.392	(1.023, 1.760)	< 0.001
Patient Care Adherence	−0.246	0.033	(0.024, 0.031)	< 0.001
Continuity of care	−0.120	0.099	(0.001, 0.207)	< 0.001
Caregiver stress	−0.076	0.104	(0.022, 0.177)	0.012
Liver transplantation	−0.059	0.206	(0.106, 0.306)	0.049

## 4 Discussion

### 4.1 Continuous Nursing Intervention Based on “Internet+” Is Beneficial to Improving the Nutritional Status of Children After Liver Transplantation

After adopting “Internet+” continuous care, the number of children at risk of malnutrition was substantially lower than in those receiving conventional continuous care. After controlling for confounding factors and collinearity, logistic regression analysis showed that patient care compliance, continuous care methods and caregiver stress had a substantial impact on the nutritional status of children after 4 weeks of care. The compliance and caregiver stress levels in the “Internet+” group were better than those in the conventional care group, but there was no significant difference in awareness of nutritional knowledge, suggesting that “Internet+” continuous care may improve the nutritional status of children by influencing the compliance of family members and the stress levels of caregivers.

Analyzing the reasons, continuity care based on the “Internet+” platform can compensate for the shortcomings of traditional continuity care. It makes full use of the Internet's features—transmission, freedom, real‐time access, interaction, sharing and openness—and provides a personalized platform for communication between nurses and patients' families. Nurses regularly transmit information in the form of text, pictures, videos and voice messages, making the nursing content more intuitive, humanized, and easier to understand (Li et al. 2021), thus making it easier for patients and their families to remember and apply. This enhances the family members' mastery of nursing knowledge and improves children's nursing compliance. Studies have shown that postoperative nutritional status is closely related to nursing compliance. A high level of compliance helps patients strictly follow dietary and medication plans, thereby improving nutritional outcomes (Su et al. 2024).

Second, the “Internet+” model overcomes geographical and practical limitations and increases interaction between nurses and patients' families. This allows for better guidance on lifestyle, medication, and rehabilitation. For existing problems, families can receive timely help through social software such as WeChat and QQ (Liang and Enhanced Recovery After Surgery Committee of the Chinese Society of Research Hospitals 2021). The real‐time feedback mechanism enables nursing staff to identify and resolve problems promptly, avoiding omissions or errors during the care process and further ensuring the effectiveness of nutritional management.

This study found that, due to the provision of real‐time monitoring and guidance through platforms such as WeChat, the observation group achieved better results. The improvement in nutritional status and compliance rate in the observation group shows that the “Internet+” platform, through personalized delivery of information (text, images, video, and audio), made the nursing content more specific and easier to follow. The extended care supported by the “Internet+” platform effectively reduced caregiver stress and negative emotions, with lower CSI and SDS scores in the observation group. This improvement is attributed to timely professional guidance and psychological support provided through the platform. The “Internet+” platform also offers a structured and standardized nursing method to ensure patients receive consistent, evidence‐based care, thereby improving the quality and effectiveness of nursing.

During the postoperative recovery phase, children who have undergone liver transplantation often face challenges in nutritional supplementation due to preoperative malnutrition, intraoperative blood loss and increased postoperative metabolic demands. PA is an important indicator reflecting the liver's synthetic function and short‐term nutritional status. In this study, the PA level in the observation group was substantially higher than in the control group, indicating that “Internet+” nursing played a positive role in improving liver metabolism and nutritional levels through scientific management. Consistent with previous studies, this study further confirms the value of continuity care in optimizing postoperative nutritional management. For example, Xie et al. found that “Internet+”‐based health guidance substantially improved nutritional compliance and related indices in elderly patients following hip replacement surgery (Xie et al. 2017). Although such studies have primarily focused on adult populations, this study is the first to systematically explore the application of this model in the postoperative care of pediatric liver transplantation.

### 4.2 The Impact of “Internet+”‐Based Continuous Nursing Intervention on the Psychological State of Caregivers

The caregiver's mental state directly affects the quality of care for the child. Negative emotions may lead to a decline in the quality of care, which in turn affects the child's recovery (Yu et al. 2021). After adopting “Internet+” continuous care, the number of complications in children was substantially lower than that in the group of children receiving conventional continuous care. After controlling for confounding factors and collinearity, logistic regression analysis showed that patient care compliance, continuous care methods, caregiver stress, and liver transplantation were all associated with the occurrence of complications in children 4 weeks later. The compliance and caregiver stress of “Internet+” continuity care were better than those of conventional continuity care, suggesting that “Internet+” continuity care may reduce the occurrence of complications in children by improving family members' compliance and reducing caregiver stress.

After being discharged from the hospital, patients still need to take medication strictly according to the doctor's instructions, avoid increasing or decreasing medication arbitrarily, and maintain a regular lifestyle. Because pediatric liver transplantation is relatively complex and the patients lack the ability to care for themselves, the caregiver plays an important role in the child's postoperative recovery (Liang and Zhu 2020). Previous studies have shown that the level of care provided by family members after pediatric liver transplantation is related to the family members' age and cultural background. Some family members lack a correct understanding of the surgery and experience a high level of care‐related stress, which leads to a higher incidence of postoperative complications (Wang 2020).

Continuous care based on “Internet+” has established a real‐time interactive channel between the patient's family and the nursing team through the WeChat platform, providing timely professional guidance and psychological support to the family, thereby reducing their anxiety and stress (Chan and Ng 2022). In addition, the nursing staff in the group check the punch‐in content daily and provide feedback, which helps to enhance the family's confidence in the nursing process and relieve their psychological burden. The relief of caregiver stress is particularly important in the management of chronic diseases in children. Previous studies have shown that the psychological burden of family members after liver transplantation mainly comes from concerns about postoperative complications and a lack of nursing knowledge. In this study, the nutritional knowledge awareness rate of family members in the observation group was substantially higher than in the control group (97.37% vs. 94.74%), which was closely related to the improvement in their mental state. Compared with previous studies, this study further verified the role of Internet‐based continuity care in providing psychological support for family members. For example, Phichaphop et al. pointed out in a study on pediatric liver transplantation that the real‐time interactive care model can substantially reduce the anxiety levels of family members and improve their confidence in caregiving (Xu et al. 2019).

Continuous nursing intervention based on “Internet+” can push relevant knowledge such as health, diet and functional exercise according to the characteristics of the children, fully mobilize the initiative of family members and attach importance to the information feedback from the children's family members, so as to understand their true thoughts in time, conduct effective and timely communication, reduce the negative emotions of the children's family members, solve the children's health problems after discharge in time, help the children's family members build confidence, thereby reducing the pressure of family members in caring for the children and improving their psychological well‐being (Xue et al. 2019; Phichaphop et al. 2020). On the other hand, taking anti‐rejection drugs correctly and regularly after surgery also has an important impact on the occurrence of postoperative complications in children. Continuous care based on “Internet+” can form a community atmosphere through clocking in and other methods and indirectly remind the family members of the children to administer medication when others clock in, ensuring the accuracy and standardization of postoperative medication (Zhang et al. 2020; Gong et al. 2020).

### 4.3 Clinical Significance and Promotion Value

The “Internet+”'‐based continuity nursing intervention substantially improved the nutritional status of children and the mental health of their families, providing an effective intervention strategy for the postoperative management of pediatric liver transplantation. First, this model makes up for the shortcomings of low follow‐up frequency and untimely communication in traditional continuity nursing through real‐time monitoring and personalized guidance; second, by establishing an information management platform, it improves the standardization and scientific nature of the nursing process and promotes the homogenization of nursing quality.

In summary, the continuous nursing intervention based on “Internet+” after pediatric liver transplantation can help improve the nutritional status of patients, reduce the pressure and negative emotions of caregivers, improve nursing compliance and reduce the incidence of postoperative complications, which is worthy of promotion and application. However, this study still has the following shortcomings: first, it is limited by the sample source, as only 76 children were selected for the study; second, the intervention effect was only analyzed 4 weeks after the intervention, but the recovery of liver function takes a long time, and continuous follow‐up should be conducted in the future to compare the long‐term benefits of the two nursing methods; third, due to the dependent variable being categorical, it is not possible to perform a mediation effect test, and it is therefore not possible to statistically prove how “Internet+” continuous nursing improves the nutritional status and complications of children after 4 weeks of care. In the future, the sample size can be further expanded, the follow‐up time increased, and a more appropriate outcome indicator measurement method selected for in‐depth discussion.

## 5 Relevance for Clinical Practice

Based on the results of this study, schools can cooperate with local communities to carry out family‐centered education. On the one hand, this can provide education to families about the importance of nutrition and mental health. On the other hand, through seminars and hands‐on practice activities, parents and caregivers can experience and understand the benefits brought by continuous care based on “Internet+,” thereby promoting the concept of “Internet+”‐based continuous care.

## Author Contributions

W.Z. conceived of the study. L.F. and Y.R. participated in its design and data analysis. L.Y. and L.Z. contributed to statistics. All authors contributed to manuscript drafting, critically reviewed, and approved the final version.

## Ethics Statement

This study was conducted in accordance with the Declaration of Helsinki and approved by the Bioethics Committee of Beijing Friendship Hospital, Capital Medical University (ethical approval number: 2023‐P2‐378‐01). We obtained signed informed consent from the participants in this study.

## Consent

The authors have nothing to report.

## Conflicts of Interest

The authors declare no conflicts of interest.

## Data Availability

Data sharing is not applicable to this article as no datasets were generated or analyzed during the current study.
